# TranCEP: Predicting the substrate class of transmembrane transport proteins using compositional, evolutionary, and positional information

**DOI:** 10.1371/journal.pone.0227683

**Published:** 2020-01-14

**Authors:** Munira Alballa, Faizah Aplop, Gregory Butler

**Affiliations:** 1 Department of Computer Science and Software Engineering, Concordia University, Montréal, Québec, Canada; 2 College of Computer and Information Sciences, King Saud University, Riyadh, Saudi Arabia; 3 School of Informatics and Applied Mathematics, Universiti Malaysia Terengganu, Malaysia; 4 Centre for Structural and Functional Genomics, Concordia University, Montréal, Québec, Canada; UMR-S1134, INSERM, Université Paris Diderot, INTS, FRANCE

## Abstract

Transporters mediate the movement of compounds across the membranes that separate the cell from its environment and across the inner membranes surrounding cellular compartments. It is estimated that one third of a proteome consists of membrane proteins, and many of these are transport proteins. Given the increase in the number of genomes being sequenced, there is a need for computational tools that predict the substrates that are transported by the transmembrane transport proteins. In this paper, we present TranCEP, a predictor of the type of substrate transported by a transmembrane transport protein. TranCEP combines the traditional use of the amino acid composition of the protein, with evolutionary information captured in a multiple sequence alignment (MSA), and restriction to important positions of the alignment that play a role in determining the specificity of the protein. Our experimental results show that TranCEP significantly outperforms the state-of-the-art predictors. The results quantify the contribution made by each type of information used.

## Introduction

Transmembrane proteins are gates that organize a variety of vital cellular functions including cell signaling, trafficking, metabolism, and energy production. It is estimated that, on average, one in every three proteins in a cell is a membrane protein [1, 2]. Any defective or misregulated membrane protein can disturb the organism’s functioning, giving rise to disease [3]. About one-half of the drug targets today are membrane proteins, such as transporters or related receptors [4]. While the amino acid sequence of many membrane proteins is available, they are among the least characterized proteins in terms of their structure and function. For example, only 3% of the structures in the Protein Data Bank [5] are transmembrane proteins.

Numerous genome projects have produced an abundance of protein sequences, many of which remain uncharacterized. Transmembrane proteins are among the least characterized proteins, because experimental characterization of their structure and function is exceptionally difficult, owing to their hydrophobic surfaces and their lack of conformational stability. Consequently, there is an urgent need for computational approaches in which the available data is used to distinguish and characterize transmembrane proteins. Yet, this area of research is still in its early stages, and the researchers are far from finding a definitive solution.

Existing tools for the annotation of transporters that predict the substrate of the transport reaction lag behind tools for other kinds of proteins such as enzymes for metabolic reactions. Most tools predict the type of substrates [6–10], chosen from a small subset of substrate types, without attempting to predict the specific substrate, or predict the family or subfamily [9, 11–13] of the protein within the transporter classification (TC) [14–16], and again without attempting to predict the specific substrate. For network modeling in systems biology [17, 18], we require tools to process the complete proteome and predict each transport reaction; this means identifying the transport protein and the specific substrate.

Many tools rely simply on homology or orthology to predict transporters. This includes metabolic network tools merlin [19], and Pantograph [20], as well as our system TransATH [21]. Amongst the tools for *de novo* prediction of substrate class, the Transporter Substrate Specificity Prediction (TrSSP) [10] claims to be the state-of-the-art.

Our previous efforts [22] for *de novo* prediction of specific substrates for sugar transporters in fungi were not successful. However, from it we learned how much depends on very few residues of the transporter; often three or so residues, and often internal to different helix transmembrane segments (TMSs) of the transporter [23]. These residues are far apart in the linear protein sequence, but are close to each other in the three-dimensional structure of the protein when integrated in the membrane. In looking forward to how we might improve on approaches that rely on the amino acid composition of the protein, we developed a roadmap, whereby the composition information would be combined with evolutionary information as captured by an MSA, and by positional information [24] about the residues responsible for determining the specificity of the transporter. This roadmap is a schema for a large number of possible algorithms due to the many choices for encoding of amino acid composition, MSA algorithms, and algorithms for specificity determining sites [25]. We also realized the importance of the alignment preserving the TMS positions, since the important residue positions seem to be located there. There are a number of such MSA algorithms [26–29].

We therefore conducted a preliminary study, which indicated that the combination of information about protein composition, protein evolution, and the specificity determining positions had a significant impact on our ability to predict the transported substrates. We chose the methodology and datasets of TrSSP [10] as our baseline, and varied this to illustrate the impact of each of the factors: compositional, evolutionary, and positional information. Our best approach, which defines the predictor we call TranCEP (an abbreviation of transporter substrate prediction using compositional, evolutionary, and positional information), involves utilizing the pair amino acid composition (PAAC) encoding scheme, the TM-Coffee [27] MSA algorithm, and the transitive consistency score (TCS) [30] algorithm for determining informative positions in the MSA, to build a suite of support vector machine (SVM) classifiers, one for distinguishing between each pair of classes of substrates.

### Background

For most of the work done on the prediction of transport proteins [3], there is no available software, so it is difficult to reproduce the work and to compare the results of different articles.

Pathway tools includes the *Transport Inference Parser* (TIP) [31], which analyses keywords in a gene annotation to assign gene-protein-reaction associations to transport reactions in MetaCyc [32].

The *Genome-Blast* (G-Blast) [33] screens proteins against all the entries in Transporter Classification Database (TCDB) [34] using Blast to retrieve the top hit, and HMMTOP [35] to determine the TMS for the query and the hit sequence. It is an integral part of a manual protocol of Saier’s lab to predict the transport proteins for a genome [36].

The Zhao Lab has developed three methods: a nearest neighbour approach [37]; TransportTP [12]; and TrSSP [10]. The nearest neighbour approach achieved a balanced accuracy of 67.0%.

The *TransportTP* [12] is a two-phase algorithm that combines homology and machine learning to predict the TC family of one or more proteins. For training and cross-validation testing, *TransportTP* used the yeast proteome. When testing, it used 10 genomes from the TransportDB database [38] of annotated prokaryote transporters. As an independent test, *TransportTP* was trained on the proteome of *A. thaliana* and then used to predict the transporters in four other plant proteomes. *TransportTP* achieved a balanced accuracy of 81.8%. Compared with the SVM-Prot classifier [39], on the five TC superfamilies and three families used by SVM-Prot, *TransportTP* achieved better recall and precision.

The *TrSSP* [10] is a web server utilized to predict membrane transport proteins and their substrate category. The substrate categories are: (1) oligopeptide (amino acid), (2) anion, (3) cation, (4) electron, (5) protein/mRNA, (6) sugar, and (7) other. *TrSSP* makes a top-level prediction of whether the protein is a transporter, or not. An SVM is applied, with the highest accuracy being reported when using amino acid index (AAindex) and position-specific scoring matrix (PSSM) features.

The *disc_function* system [11] uses amino acid composition and neural networks to discriminate channels/pores, electrochemical and active transporters, with an accuracy of 68%. When augmented with PSSM profiles and amino acid physicochemical properties it gained 5–10% in discrimination accuracy [13]. They also considered six major families in TCDB [13] with an average accuracy of 69%.

The *TTRBF* [7] considers four major classes of substrates: (1) electron, (2) protein/mRNA, (3) ion, and (4) other. It is an ensemble system combining amino acid composition, dipeptide composition, physicochemical properties, PSSM profiles and radial basis function (RBF) networks.

*Schaadt et al*. [6] used amino acid composition, characteristics of amino acid residues and conservation to detect transporters based on four classes of substrate: (1) amino acid, (2) oligopeptide, (3) phosphate, and (4) hexose. The number of characterized transporters in *A. thaliana* for the four substrates was from 13 to 17. They constructed a vector for each protein using various types of amino acid composition, and used Euclidean distance from the query protein’s vector to the known vectors to rank the substrate categories. They found that the PAAC performed as well as the more complicated features, yielding an accuracy of over 90%.

*Schaadt and Helms* [8] compared the similarity of transporters in the TCDB and annotated transporters in *A. thaliana* using amino acid composition and classified the proteins into three families. By distinguishing the amino acid composition of TMS and non-TMS regions, they could classify four different families with an accuracy of 80%.

*Barghash and Helms* [9] performed a comparison of three different approaches (homology, HMMER, MEME) to predict the substrate category and predicting TC family. They used four substrate categories, namely metal ions, phosphate, sugar, and amino acid; and the 29 TC families with the most examples. The datasets were from *E. coli*, *S. cerevisiae*, and *A. thaliana*, consisting of the 155, 158 and 177 proteins, respectively, that had both a substrate annotation and TC family annotation that were experimentally determined.

The *merlin* [19] system for the reconstruction of metabolic networks handles eukaryote genomes, and includes the determination of transport gene-protein-reaction associations, as well as the localization of reactions across a number of cellular compartments: mitochondrion, endoplasmic reticulum (ER), and Golgi apparatus. In *merlin*, transport proteins are predicted based on the existence of TMS as predicted by TMHMM, and by similarity to entries in the TCDB using the Smith-Waterman algorithm [40]. The association of transport reactions and specific substrates for the predicted transport proteins were originally taken from a manually curated database of some 4,000 TCDB entries originally. It now incorporates *Transport Reactions Annotation and Generation* (TRIAGE) [41], with information for 5,495 TCDB entries. The *merlin* software is available as open source Java code (http://www.merlin-sysbio.org).

The *Pantograph* [20] is designed for the metabolic pathway reconstruction of yeasts such as *Yarrowia lipolytica* [42], which accumulates lipids in the peroxisome of the cell. It specifically models the cellular components and the transport across the membranes in a reference template, called the *scaffold model*. The Pantograph method relies on a database of profile HMMs for fungal protein families and their annotations, which is maintained at Génolevures in Bordeaux. The Pantograph algorithm first assigns gene-protein-reaction associations, and then decides which compartments and reactions to include in the draft model based on these associations. The scaffold model, which is the reference template for Pantograph, was manually curated to include 421 transport reactions. The associated transport protein families of orthologs were manually identified for Génolevures collection. The Pantograph software, written in Python, is available at http://pathtastic.gforge.inria.fr/. The distribution includes the scaffold model in Systems Biology Markup Language (SBML).

The *Transporters via Annotation Transfer by Homology* (TransATH) [21] is a system that automates Saier’s protocol based on sequence similarity. The TransATH includes the computation of subcellular localization and improves the computation of TMSs. The parameters of TransATH are chosen for optimal performance on a gold standard set of transporters and non-transporters from *S. cerevisiae*. A website http://transath.umt.edu.my for TransATH is available for use.

The *SCMMTP* [43] has a novel scoring card method (SCM) that utilizes the dipeptide composition to identify putative membrane transport proteins. The SCMMTP method first builds an initial matrix of 400 dipeptides and uses the difference between compositions of positives and negatives as an initial dipeptide scoring matrix. This matrix is then optimized using a genetic algorithm. *SCMMP* achieved an overall accuracy of 76.11% and Matthews correlation coefficient (MCC) of 0.47 on the independent dataset.

*Li et al* [44] tool uses an SVM model to predict substrate classes of transmembrane transport proteins by integrating features from PSSM, amino acid composition, biochemical properties and gene ontology (GO) [45] terms. The tool achieved an overall accuracy of 80.45% on the independent dataset.

## Materials and methods

### Datasets

We used the same training dataset and test dataset as TrSSP by Mishra *et al*. [10] which are available at http://bioinfo.noble.org/TrSSP. This benchmark dataset was collected from the Swiss-Prot database (release 2013-03). The dataset initially contained 10,780 transporter, carrier, and channel proteins that were well characterized at the protein level and had clear substrate annotations. Then, Mishra *et al*. removed the transporters with more than two substrate specificities, sequences with biological function annotations based solely on sequence similarity, and sequences with greater than 70% similarity. Mishra *et al.’s* final transporter dataset contains 900 transporters divided into seven substrate classes: *amino acid*, *anion*, *cation*, *electron*, *protein/mRNA*, *sugar*, and *other*. The latter refers to transporters that do not belong to any of the previous six classes. Table 1 shows the dataset details with the size of each class.

**Table 1:** The dataset.

Substrate class	Training dataset	Test dataset
Amino acid	70	15
Anion	60	12
Electron	60	10
Cation	260	36
Protein/mRNA	70	15
Sugar	60	12
Other	200	20
**Total**	780	120

### Databases

We used the Swiss-Prot database when searching for similar sequences. When constructing MSAs, we used TM-Coffee [27] with the UniRef50-TM database, which consists of the entries in UniRef50 that have keyword *transmembrane*.

The TrSSP dataset was derived from the Swiss-Prot database, so we made sure to remove all entries in dataset from the two databases that we used.

### Algorithm

**Fig 1:**
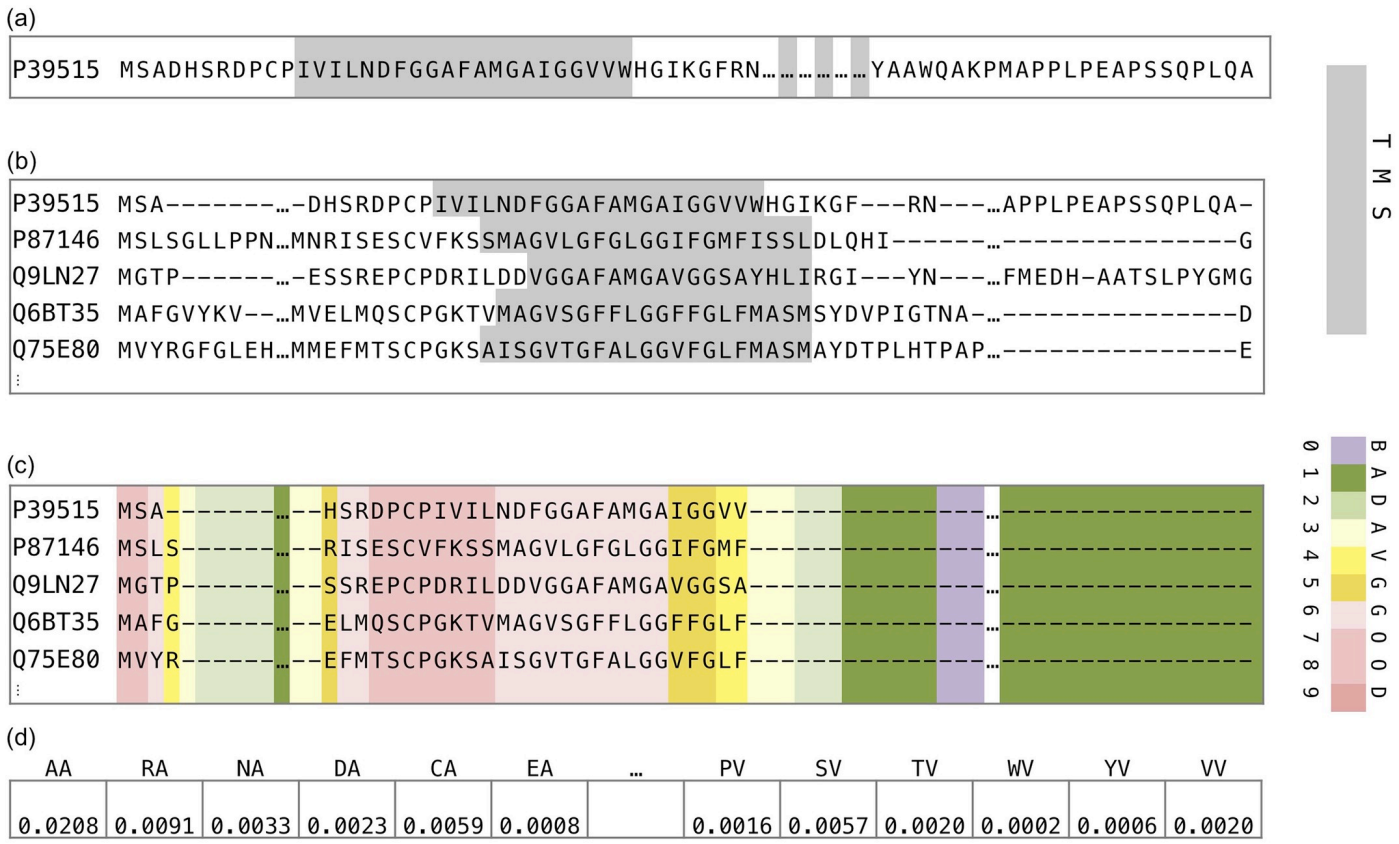
Example of steps of TranCEP. The figure illustrates the steps of TranCEP. Note that we abbreviated the middle section of the sequence. Part (a) shows the sequence the four TMSs in gray shading. Part (b) shows an MSA constructed by TM-Coffee. Gray shading indicates a TMS. Part (c) shows the color coding of the reliability index of each column as determined by TCS, and shows gaps in unreliable columns in the filtered MSA. Part (d) shows a 400-dimensional PAAC vector from the filtered MSA.

Fig 1 illustrates the steps of TranCEP. The sequence (a) in this case, according to TM-Coffee, has four TMSs, as shown by the gray shading. The example focuses on the first TMS, and abbreviates the middle section of the sequence. Part (b) shows an MSA conserving the TMS structure constructed by TM-Coffee, where the gray shading indicates the TMS location. Part (c) shows the colour coding of the reliability index of each column as determined by TCS, and shows how gaps replace unreliable columns in the filtered MSA. Part (d) shows a part of the 400-dimensional vector of dipeptide frequencies (PAAC) from the filtered MSA.

The template for combining evolutionary, positional, and compositional information is presented in Algorithm 1. In this work, we used TM-Coffee to compute the MSA that conserves the TMSs, and TCS to determine a reliability index for each position (column) in the MSA. We experimented with three composition schemes: amino acid composition (AAC), pairwise amino acid composition (PAAC), and pseudo-amino acid composition (PseAAC), as well as the optional use of TM-Coffee (TMC) and the transitive consistency score (TCS).

**Algorithm 1**. **Template for constructing the composition vector**.

 **function** comp_vec(seq *s*)

  // *Evolutionary (E) Step, optional*

  Construct an MSA from *s*

  // *Positional (P) Step, optional*

  Determine the informative positions (columns) in the MSA

  Filter the uninformative positions from the MSA

  // *Composition (C) Step, mandatory*

  **return** vector encoding composition of the filtered MSA

 **end function**

A template for the algorithm showing the role of evolutionary (E), positional (P), and composition (C) information. Note that the use of evolutionary (E) and positional (P) is optional; and that if positional (P) information is used then it requires evolutionary (E) information in the form of Multiple Sequence Alignment (MSA). Note that if Step (E) is not done, then Step (C) encodes the sequence *s*. Note that if Step (E) is done but Step (P) MSA is not done, then Step (C) encodes the MSA.

Algorithm 2 shows the composition vectors being used to build a set of classifiers; SVM classifiers in this case. Algorithm 3 shows the prediction algorithm.

**Algorithm 2**. **Build SVM classifiers**.

**Require**: a training set *T* of sequences labelled with classes *C*_1_, …, *C*_*n*_

**Ensure**: a set of SVM’s *svm*(*i*, *j*) distinguishing class *C*_*i*_ and *C*_*j*_

 **procedure** Build_SVMs(*T*: set of seq; *svm*: set of SVM)

  **for all** seq *s* in *T* **do**

   *v*(*s*) ← COMP_VEC(*s*)

  **end for**

  **for all** pair (*C*_*i*_, *C*_*j*_) of classes **do**

   *svm*(*i*, *j*) ← SVM.build({*v*(*s*): *s* ∈ *T* ∩ (*C*_*i*_ ∪ *C*_*j*_)})

  **end for**

 **end procedure**

The algorithm to build a set of SVM classifiers to distinguish between each pair of classes in the training set. The actual construction of each SVM was done by an SVM package’s build function.

**Algorithm 3**. **Prediction**.

**Require**: test sequence *s*

**Require**: a set of SVM’s *svm*(*i*, *j*) distinguishing classes *C*_*i*_ and *C*_*j*_

**Ensure**: result is predicted class *C*_*p*_

 **function** predict_class(seq *s*)

  *v* ← COMP_VEC(*s*)

  **for all** pair (*C*_*i*_, *C*_*j*_) of classes **do**

   *c*(*i*, *j*) ← *svm*(*i*, *j*) applied to *v*

  **end for**

  *p* ← mode of *c*(*i*, *j*) for all pairs (*i*, *j*)

  **return** *C*_*p*_

 **end function**

The prediction algorithm that applies each of the SVMs, and takes the class that is predicted most often by the set of SVMs.

### Encoding amino acid composition

The properties of the amino acids at each position in the protein sequence can be encoded into vectors that summarize the overall composition of the protein. Three approaches to encoding amino acid composition were implemented in this study: *AAC*, *PAAC*, and *PseAAC*.

The *amino acid composition* (AAC) is the normalized occurrence frequency of each amino acid. The fractions of all 20 natural amino acids are calculated as:

$$\begin{array}{c} c_{i}=\frac{F_{i}}{L}\;i=\left(1,2,3,\ldots 20\right) \end{array}$$ (1)

where *F*_*i*_ is the frequency of the *i*^*th*^ amino acid in sequence P, and *L* is the length of the sequence. The AAC is represented as a vector of size 20:

$$\begin{array}{c} AAC\left(P\right)=[c_{1},c_{2},c_{3},\ldots ,c_{20}] \end{array}$$ (2)

where *c*_*i*_ is the composition of *i*^*th*^ amino acid.

The *pair amino acid composition* (PAAC) is the normalized frequency of each pair of amino acids. The PAAC is calculated as

$$\begin{array}{c} d_{i,j}=\frac{F_{i,j}}{L-1}\;i,j=\left(1,2,3,\ldots 20\right) \end{array}$$ (3)

where *F*_*i*,*j*_ is the frequency of the *i*^*th*^ and *j*^*th*^ amino acids as a pair (dipeptide) adjacent to each other in the sequence P, and *L* is the length of the sequence. The PAAC is represented as a vector of size 400 as follows:

$$\begin{array}{c} PAAC\left(P\right)=[d_{1,1},d_{1,2},d_{1,3},\ldots ,d_{20,20}] \end{array}$$ (4)

where *d*_*i*,*j*_ is the dipeptide composition of the *i*^*th*^ and *j*^*th*^ amino acid.

The *pseudo-amino acid composition* (PseAAC) [46] combines the 20 components of AAC with a set of *sequence order correlation factors* that incorporates some biochemical properties. Given a protein sequence *P* = *R*_1_*R*_2_*R*_3_*R*_4_…*R*_*L*_ of length *L*, a set of descriptors called sequence order-correlated factors are defined as:

$$\begin{array}{c} \left\{ \begin{array}{c} \theta_{1}=\frac{1}{L-1}\sum_{i=1}^{L-1}\Theta \left(R_{i},R_{i+1}\right)\\ \theta_{2}=\frac{1}{L-2}\sum_{i=1}^{L-2}\Theta \left(R_{i},R_{i+2}\right)\\ \theta_{3}=\frac{1}{L-3}\sum_{i=1}^{L-3}\Theta \left(R_{i},R_{i+3}\right)\\ .\\ .\\ .\\ \theta_{\lambda }=\frac{1}{L-\lambda }\sum_{i=1}^{L-\lambda }\Theta \left(R_{i},R_{i+\lambda }\right) \end{array}\right. \end{array}$$ (5)

The parameter λ is chosen such that (λ < *L*). A correlation function is given by:

$$\begin{array}{c} \Theta \left(R_{i},R_{j}\right)=\frac{1}{3}\{{\left[H_{1}\left(R_{j}\right)-H_{1}\left(R_{i}\right)\right]}^{2}+{\left[H_{2}\left(R_{j}\right)-H_{2}\left(R_{i}\right)\right]}^{2}+{\left[M\left(R_{j}\right)-M\left(R_{i}\right)\right]}^{2}\} \end{array}$$ (6)

where *H*_1_(*R*_*i*_) is the hydrophobicity value, *H*_2_(*R*_*i*_) is the hydrophilicity value, and *M*(*R*_*i*_) is the side chain mass of the amino acid *R*_*i*_. Those quantities are converted from their original values. For example, for hydrophobicity, *H*_1_(*R*) is derived from the average hydrophobicity value $H_{1}^{{}^{\circ}}\left(R_{i}\right)$, as follows:

$$\begin{array}{c} H_{1}\left(R_{i}\right)=\frac{H_{1}^{{}^{\circ}}\left(R_{i}\right)-\frac{1}{20}\sum_{k=1}^{20}H_{1}^{{}^{\circ}}\left(R_{k}\right)}{\sqrt{\frac{\sum_{y=1}^{20}{\left[H_{1}^{{}^{\circ}}\left(R_{y}\right)-\frac{1}{20}\sum_{k=1}^{20}H_{1}^{{}^{\circ}}\left(R_{k}\right)\right]}^{2}}{20}}} \end{array}$$ (7)

The original hydrophobicity value $H_{1}^{{}^{\circ}}\left(R_{i}\right)$ is taken from Tanford [47]. The original hydrophilicity value $H_{2}^{{}^{\circ}}\left(R_{i}\right)$ for the amino acid *R*_*i*_ is taken from Hopp and Woods [48].

PseAAC is represented as a vector of size (20 + λ) as follows:

$$\begin{array}{c} PseAAC\left(P\right)=[s_{1},\ldots ,s_{20},s_{21},\ldots ,s_{20+\lambda }] \end{array}$$ (8)

where *s*_*i*_ is the pseudo-amino acid composition

$$\begin{array}{c} s_{i}=\{\begin{array}{cc} \frac{f_{i}}{\sum_{r=1}^{20}f_{r}+\omega \sum_{j=1}^{\lambda }\theta_{j}} & 1\leq i\leq 20\\ \frac{\omega \theta_{i-20}}{\sum_{r=1}^{20}f_{r}+\omega \sum_{j=1}^{\lambda }\theta_{j}} & 20<i\leq 20+\lambda \end{array} \end{array}$$ (9)

where *f*_*i*_ is the normalized occurrence frequency of the *ith* amino acid in the protein sequence, *θ*_*j*_ is the *j*^*th*^ sequence order-correlated factor calculated from Eq 5, and *ω* is a weight factor for the sequence order effect. The weight factor *ω* puts weight on the additional PseAAC components with respect to the conventional AAC components. Any value from 0.05 to 0.7 can be selected for the weight factor. The default value given by Chou [46] is 0.05, which we selected in this study.

### Multiple sequence alignment

We adopted the *MSA-AAC* approach [6] that combines amino acid composition with the evolutionary information available from the MSA. This is done by first retrieving homologous sequences of each protein sequence in the dataset, then building an MSA for the corresponding protein, and then taking the counts for computing the composition information using all the residues in the MSA.

In [6], the researchers only utilized the *AAC* encoding, whereas we also applied the approach to the *PAAC* and *PseAAC* encodings. Another difference was that we made use of TM-Coffee [27] (Version-11.00.8cbe486) to compute alignments, rather than ClustalW [49] as done in [6], because we felt it was important to align the TMSs.

Other differences included searching the Swiss-Prot [50] database and retrieving a maximum of 120 homologous sequences, instead of searching the non-redundant database nr and retrieving 1,000 sequences. This was done to make the computational time more manageable, because the TM-Coffee algorithm requires more memory usage and execution time.

Furthermore, all exact hits of the test sequences were removed from the Swiss-Prot and UniRef50-TM databases to maintain independence between the MSA and the test data.

Our alignment command was the following:

t_coffee mysequences. fasta −mode psicoffee \
−protein_db uniref50 −TM \
−template_file PSITM

where mysequences.fasta is the file that contains the 120 similar sequences retrieved by Blast search on the Swiss-Prot database.

### Positional information

In order to focus on those positions in the protein that determine specificity, we needed a method to determine those positions, and then to filter our MSA. The MSA was filtered by setting the entries for all other positions to null, that is, the symbol ‘-’ so that it was ignored when gathering counts for the amino acid composition.

We applied the *transitive consistency score* (TCS) algorithm [30] to the alignment to determine the informative positions. The TCS is a scoring scheme that uses a consistency transformation to assign a reliability index to every pair of aligned residues, to each individual residue in the alignment, to each column, and to the overall alignment. This scoring scheme has been shown to be highly informative with respect to structural predictions based on benchmarking databases. The reliability index ranges from 0 to 9, where 0 is extremely uncertain and 9 is extremely reliable. Columns with a reliability index of below 4 were removed using the following command:

t_coffee −infile myMSA. aln −evaluate \
−output tcs_column_filter4. fasta

where myMSA.aln is the MSA file, tcs_column_filter4.fasta is the filtered file in FASTA format.

### Training

Following TrSSP [10], we used a multi-class SVM with RBF kernel, as implemented in the R e1071 library version 1.6-8, using a one-against-one approach in which 21 = (7 × 6)/2 binary classifiers were trained. The predicted class was determined through a voting scheme in which all the binary classifiers were applied and the class that got highest number of votes was the result. Both the cost and gamma parameters of the RBF kernel were optimized by performing a grid search using the *tune* function in the library (range of cost: 2 … 16, range of gamma: 2e-5 … 1).

### Methods

We adopted three approaches to encode the amino acid composition: *AAC*, *PAAC*, as done by TrSSP [10], and *PseAAC*. This was followed by training using SVM to form the prediction methods **AAC**, **PAAC**, and **PseAAC** respectively.

By combining the amino acid composition and the evolutionary information obtained using TM-Coffee, followed by SVM, we implemented the prediction methods: **TMC-AAC**, **TMC-PAAC**, and **TMC-PseAAC** respectively.

Filtering was incorporated by applying TCS after TM-Coffee, then computing the amino acid composition vectors, and applying SVM to implement the prediction methods: **TMC-TCS-AAC**, **TMC-TCS-PAAC**, and **TMC-TCS-PseAAC** respectively.

The method of **TranCEP** is **TMC-TCS-PAAC**, the method with the best performance during cross-validation.

### Performance measurement

Four statistical measures were considered to measure the performance:

**sensitivity** which is the proportion of positives that are correctly identified:
  $$\begin{array}{c} Sensitivity=\frac{TP}{TP+FN} \end{array}$$ (10)
**specificity** which is the proportion of negatives that are correctly identified:
  $$\begin{array}{c} Specificity=\frac{TN}{TN+FP} \end{array}$$ (11)
**accuracy** which is proportion of correct predictions made amongst all the predictions:
  $$\begin{array}{c} Accuracy=\frac{TP+TN}{TP+FN+TN+FP} \end{array}$$ (12)
**Matthews correlation coefficient (MCC)** which is a single measure taking into account true and false positives and negatives:
  $$\begin{array}{c} MCC=\frac{\left(TP\times TN-FP\times FN\right)}{\sqrt{\left(TP+FP)\times (TP+FN)\times (TN+FP)\times (TN+FN\right)}} \end{array}$$ (13)

where *TP* is the number of true positives, *TN* is the number of true negatives, *FP* is the number of false positives, and *FN* is the number of false negatives.

We used the MCC because it is less influenced by imbalanced data and is arguably the best single assessment metric in this case [51–53]. The MCC value ranges from 1 to −1, where 1 indicates a perfect prediction; 0 represent no better than random; and −1 implies total disagreement between prediction and observation. A high MCC value means the predictor has high accuracy on both positive and negative classes, and also low misprediction on both.

When dealing with multiclass classification, it is often desirable to compute a single aggregate measure that reflects the overall performance. There are two methods to compute the overall performance, namely *micro-averaging* and *macro-averaging* [54]. Macro-averaging computes a simple average performance of individual classes’ performances. Micro-averaging computes an overall performance by globally counting the total true positives, false negatives and false positives. Depending on the class distribution the difference between the two methods can be large. Macro-averaging gives equal weight to each class, whereas micro-averaging gives equal weight to each individual classification decision [54]. The overall accuracy of the tool is often calculated as the fraction of the correct predictions by the total number of predictions as follows:

$$\begin{array}{c} Accuracy_{overall}=\sum_{k=1}^{K}\frac{TP_{k}}{N} \end{array}$$ (14)

where *TP*_*k*_ is the number of true positives in class k, *K* is the number of different classes, and *N* is the total number of predictions.

Another way to compute the accuracy is to take the macro-average accuracy of the individual classes:

$$\begin{array}{c} Accuracy_{macro}=\frac{1}{K}\sum_{k=1}^{K}Accuracy_{k} \end{array}$$ (15)

where *Accuracy*_*k*_ is the accuracy of class k, and *K* is the number of different classes.

Similarly, the overall MCC is calculated in terms of the confusion matrix *C* of dimension *K* × *K* [55]:

$$\begin{array}{c} {MCC}_{overall}=\frac{\sum_{k}\sum_{l}\sum_{m}C_{kk}C_{lm}-C_{kl}C_{mk}}{\sqrt{\sum_{k}\left(\sum_{l}C_{kl}\right)\left(\sum_{k^{\prime }|k^{\prime }\neq k}\sum_{l^{\prime }}C_{k^{\prime }l^{\prime }}\right)}\sqrt{\sum_{k}\left(\sum_{l}C_{lk}\right)\left(\sum_{k^{\prime }|k^{\prime }\neq k}\sum_{l^{\prime }}C_{l^{\prime }k^{\prime }}\right)}} \end{array}$$ (16)

Or as a macro-average MCC:

$$\begin{array}{c} MCC_{macro}=\frac{1}{K}\sum_{k=1}^{K}MCC_{k} \end{array}$$ (17)

where *MCC*_*k*_ is the accuracy of class k, and *K* is the number of different classes.

Because the number of samples in each class of the dataset is imbalanced, we used the overall accuracy as in Eq 14 and overall MCC as in Eq 16 to evaluate and compare different methods. It is explicitly stated when the macro-average was used.

### Experiments

The performance of each method was determined using five-fold cross-validation whereby the training dataset was randomly partitioned into five equal sized sets. A single set was kept as the validation data and the remaining four sets were used to train the SVM model. This model was then tested using the validation set. The cross-validation process was repeated four times; where each of the sets was used once as the validation data. The performance of each model was averaged to produce a single estimation and the variation in performances was captured by computing the standard deviation. In addition, we ran leave-one-out cross-validation (LOOCV) on different methods to evaluate their robustness and compared the results with those obtained by five-fold cross-validation. Furthermore, the independent dataset was used to test the final models. The data in the independent dataset was not used, nor considered, during the training process and completely unknown to the different models.

The performance of TranCEP on the test dataset was compared to the performance of TrSSP, as reported in their paper [10].

### Statistical analysis

In this analysis, the average number of informative residues, as determined by TCS scores, in different segments of a protein sequence was computed. For each substrate class, pairwise comparisons between means of important positions in different segments were performed. Since the sample size of each substrate class is >30, based on the the central limit theorem [56], the Student t-test (two tailed, paired) was applied. The difference was considered statistically significant when the Student t-test significance level P (P-value) was less than 0.0001.

## Results and discussion

### Methods evaluation

**Fig 2:**
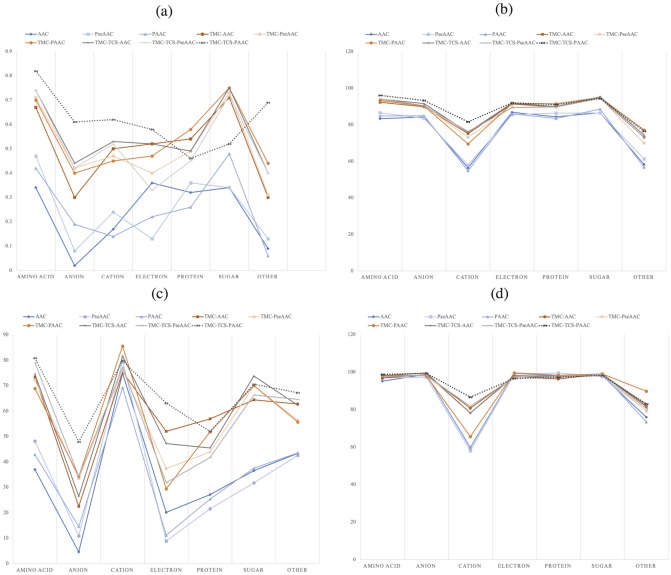
Performance of different methods. (a) MCC, (b) accuracy, (c) sensitivity, and (d) specificity. The dotted line represent the performance of TranCEP, which is TCS-TMC-PAAC.

The performance of the nine methods was evaluated using five-fold cross-validation. Table 2 presents the overall accuracy and MCC of the SVM models for the nine methods, sorted from the best to the worst MCC. Details of the performance for each method is available in S1 File; the comparison between the different methods among the seven classes in MCC, accuracy, sensitivity, and specificity is presented in Fig 2.

**Table 2:** Overall cross-validation performance of the methods.

Method	Accuracy	MCC
TMC-TCS-PAAC	69.23± 06.1	0.63±0.122
TMC-TCS-AAC	65.13± 03.2	0.58±0.125
TMC-PAAC	63.33± 04.4	0.51±0.129
TMC-AAC	63.08± 03.6	0.50±0.092
TMC-TCS-PseAAC	63.30± 05.5	0.49±0.129
TMC-PseAAC	61.79± 02.9	0.49±0.131
PseAAC	47.69± 02.4	0.27±0.104
PAAC	45.89± 03.1	0.25±0.101
AAC	45.38± 03.3	0.22±0.099

For each method, the table presents accuracy and MCC as *m*±*d*, where *m* is the mean and *d* is the standard deviation across the five runs of the cross-validation.

All the SVM models that utilized evolutionary data notably performed better than the SVM models that did not. In addition, the top two models, **TMC-TCS-PAAC** and **TMC-TCS-AAC**, integrated positional and evolutionary information. The highest MCC was obtained by **TMC-TCS-PAAC**, which is the method chosen for our predictor **TranCEP**. This method incorporates the use of PAAC with evolutionary data in the form of MSA with positional information, in which columns that have a reliability below 4 are filtered out. We found that the performance peaked using this threshold and started to decline when filtering columns with a higher reliability index. The **TMC-TCS-PAAC** method yielded an overall MCC of 0.63.

The detailed performance of **TMC-TCS-PAAC** on five-fold cross-validation is presented in Table 3, The MCC was 0.82, 0.61, 0.62, 0.58, 0.46, 0.52, and 0.69 for *amino acid*, *anion*, *cation*, *electron*, *protein/mRNA*, *sugar*, and *other*, respectively.

**Table 3:** Cross validation performance for TMC-TCS-PAAC.

Class	Specificity	Sensitivity	Accuracy	MCC
Amino acid	98.82±00.4	80.84±05.4	96.06±01.7	0.82±0.042
Anion	99.30±00.7	47.89±19.9	93.33±01.4	0.61±0.119
Cation	86.66±02.2	79.70±04.3	81.50±03.9	0.62±0.081
Electron	96.58±03.0	63.13±11.8	92.01±01.9	0.58±0.098
Protein	97.21±01.8	52.00±08.7	90.93±03.1	0.46±0.036
Sugar	98.19±01.9	70.51±10.5	94.39±02.1	0.52±0.192
Other	83.02±03.5	67.23±19.8	76.43±06.8	0.69±0.178
**Overall**			69.23±06.1	0.63±0.122

The table presents the five-fold cross-validation results for TranCEP, which is TCS-TMC-PAAC. For each substrate class, the table indicates specificity, sensitivity, accuracy and MCC as *m*±*d*, where *m* is the mean and *d* is the standard deviation across the five runs of the cross validation.

**Fig 3:**
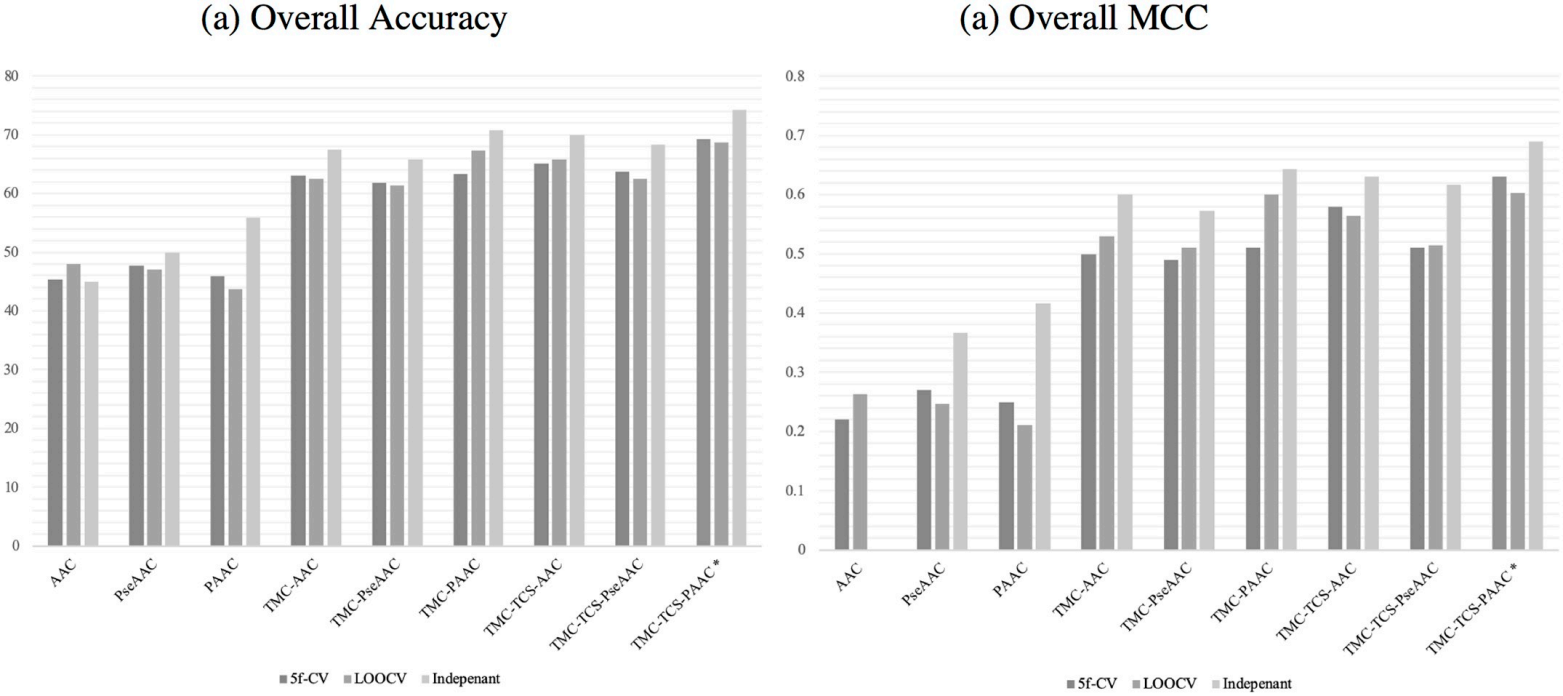
Methods overall performance. (a) overall accuracy, and (b) overall MCC. The asterisk (*) symbol refers to the performance of TranCEP, which is TCS-TMC-PAAC.

The performance evaluation using LOOCV and independent testing prediction yielded similar patterns (available in S2 and S3 Files, respectively). A comparison of the results obtained by five-fold cross-validation, LOOCV, and the independent testing is presented in Fig 3.

Table 4 shows the confusion matrix for TranCEP when run on the independent test set. Most of the confusion occurred between a substrate class and the class *other*.

**Table 4:** Confusion matrix for TranCEP.

**Actual\Predicted**	Amino acid	Anion	Cation	Electron	Protein	Sugar	Other	**Total**
Amino acid	9	0	2	0	0	0	4	15
Anion	1	7	1	0	0	0	3	12
Cation	0	0	34	0	1	0	1	36
Electron	0	0	1	8	0	0	1	10
Protein	0	0	3	0	10	0	2	15
Sugar	0	1	0	0	0	8	3	12
Other	1	3	2	0	0	1	13	20

The table presents the number of proteins in an actual substrate class that are predicted by TranCEP to fall in each substrate class. The independent test set is used.

### Comparison

Table 5 compares the performance of TranCEP to TrSSP [10] on the independent test set. TranCEP scored higher for all the substrate classes in terms of accuracy, specificity, and MCC. For the true positive rate as measured by sensitivity, TranCEP performed better on the *cation* and *other* classes, and TrSSP either matched or outperformed TranCEP on the other five classes. In TranCEP, the overall accuracy was computed as in Eq 14, and the MCC as in Eq 16. The performance of TrSSP was computed as macro-average accuracy and macro-average MCC. When we adopted the same method as TrSSP, the macro-average accuracy of TranCEP is 91.23% and the macro-average MCC is 0.69. Interestingly, the MCC score was not effected when using the overall and the macro-average, which further highlights its robustness when dealing with imbalanced data.

**Table 5:** Comparing TranCEP and TrSSP.

Class	Specificity		Sensitivity		Accuracy		MCC	
	TrSSP	TranCEP	TrSSP	TranCEP	TrSSP	TranCEP	TrSSP	TranCEP
Amino acid	82.42	98.10	93.33	60.00	83.33	91.75	0.49	**0.66**
Anion	69.05	96.30	75.00	58.33	69.44	90.82	0.23	**0.56**
Cation	74.31	89.29	75.00	94.44	74.44	89.00	0.41	**0.78**
Electron	91.78	100.0	80.00	80.00	91.11	97.80	0.50	**0.88**
Protein	82.42	99.07	93.33	66.67	83.33	93.68	0.49	**0.75**
Sugar	76.79	99.07	91.67	66.67	77.78	94.68	0.38	**0.74**
Other	73.13	86.00	60.00	65.00	71.67	80.91	0.23	**0.44**
**Overall**					NA	74.17	NA	**0.69**
**Macro-average**					78.88	91.23	0.41	**0.69**

The table presents the performance of TranCEP built with the complete training set and run on the independent test set. The corresponding results for TrSSP are taken from their original paper. The table shows specificity, sensitivity, accuracy and MCC for each of the seven substrate types; the overall accuracy and MCC, and the macro-average accuracy and MCC. The overall accuracy was calculated by TranCEP as the proportion of correct predictions divided by the total number of predictions, and the overall MCC was calculated from the confusion matrix as in Eq 16. The TrSSP performance was calculated as the average across the seven classes (macro-average); if we adopt the same method the macro-average accuracy of TranCEP is 91.23% and the macro-average MCC is 0.69. NA: not available.

Overall, TranCEP obtained an MCC of 0.69 which is 68% higher than that of TrSSP.

### Impact of factors

Table 6 shows the impact of evolutionary information and positional information on the overall MCC from the five-fold cross-validation.

**Table 6:** Impact of factors on performance.

Encoding	MCC			TMC-X to X		TMC-TCS-X to X		TMC-TCS-X to TMC-X	
X	X	TMC-X	TMC-TCS-X	Delta	Percent	Delta	Percent	Delta	Percent
AAC	0.22	0.50	0.58	0.28	127%	0.36	164%	0.08	16%
PAAC	0.25	0.51	0.63	0.26	104%	0.38	152%	0.12	24%
PseAAC	0.27	0.49	0.49	0.22	81%	0.22	81%	0.00	0%
**Average**				0.25	104%	0.32	132%	0.07	13%

The table notes the difference in MCC, the delta, and the percentage improvement in MCC, the percent, of the cross-validation performance for the introduction of evolutionary information using TM-Coffee, and positional information using TCS. The use of evolutionary information in the form of an MSA on the composition encodings **AAC**, **PAAC**, and **PseAAC** improved the MCC by an average of 104%. The further use of positional information by filtering out the unreliable columns from the MSA boosted the MCC of the composition encodings by an average of 132%.

The use of evolutionary information in form of MSA on the composition encodings **AAC**, **PAAC**, and **PseAAC** improved the MCC by an average of 104% with the highest improvement being on **AAC** by 127%. The further use of positional information by filtering out the unreliable columns from the MSA showed an average improvement of 132% to the baseline compositions. The impact of positional information over that already achieved by evolutionary information was an average of 13%. The highest impact is on **TMC-PAAC**, where the MCC improved by 24% in **TMC-TCS-PAAC**.

It is difficult to isolate the exact residues that are key to inferring the substrate class; the results suggest that evolutionary information, obtained by MSA, is the main source of achieving high prediction performance. In addition, the TCS informative positions (with TCS score ≥ 4) can help to filter out the unnecessary noise and get a clearer signal to further improve the prediction. Using TCS informative positions filtered out an average of (35% ± 7%) of the sequence. However, when we tried to filter out more positions (by taking a stricter the TCS score cut-off), the performance started to deteriorate.

**Fig 4:**
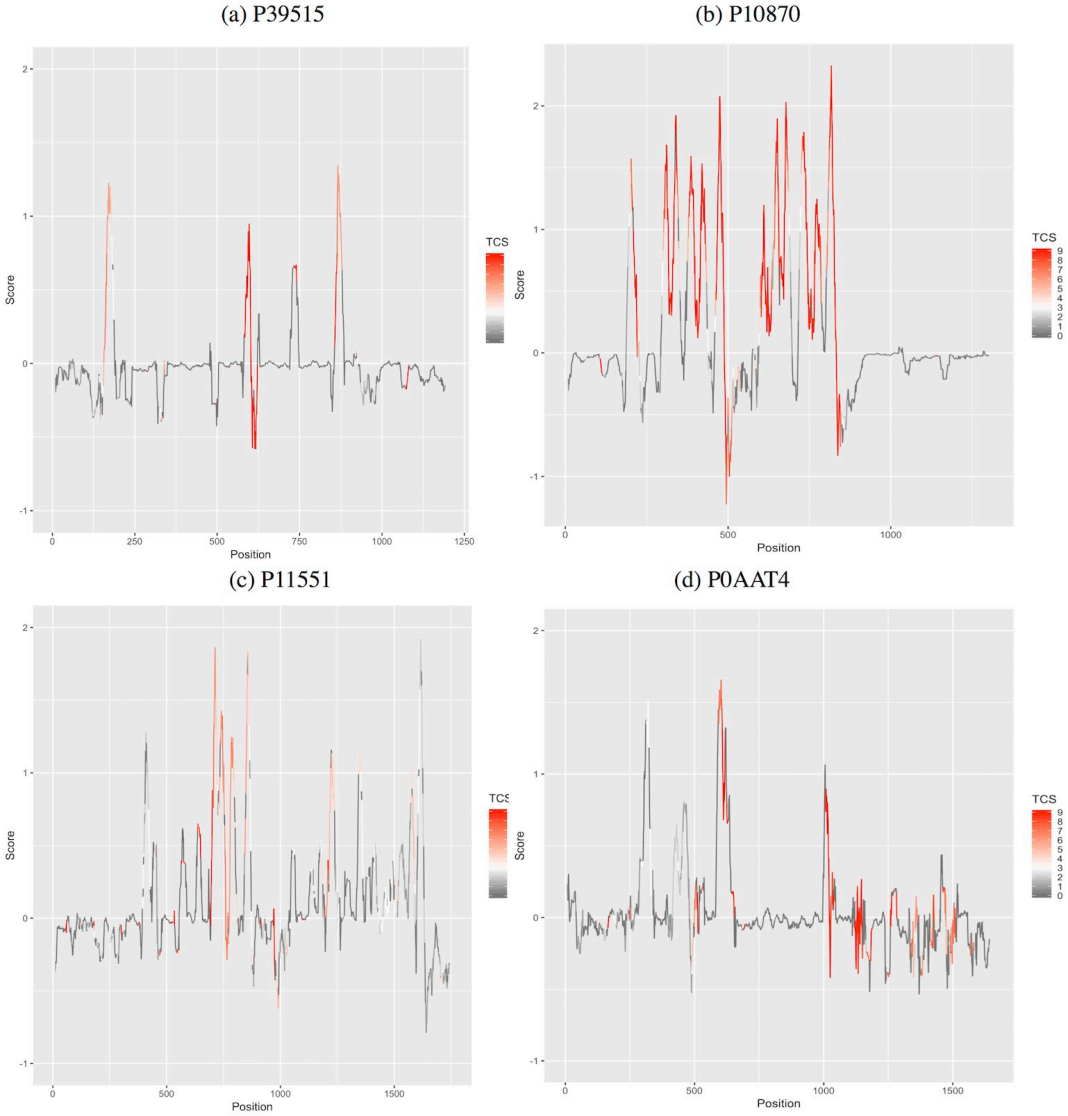
Average Kyte-Doolittle hydropathy of the MSA with TCS scores. The figure indicates that the columns highlighted in red are informative and used by TranCEP. The TranCEP considers a column to be informative if it has a TCS score of at least 4 (shades of red), and filters out the other columns (gray and white). In (a) P39515 contains 158 residues, and the alignment of P39515 with other homologous sequences has 1203 columns, only 128 of them are informative (highlighted in shades of red). In (b) P10870 contains 884 residues, and the alignment of P10870 with other homologous sequences has 1312 columns, only 402 of them are informative. In (c) P11551 contains 438 residues, and the alignment of P11551 with other homologous sequences has 1753 columns, only 343 of them are informative. In (d) P0AAT4 contains 415 residues, and the alignment of P0AAT4 with other homologous sequences has 1653 columns, only 274 of them are informative.

To visualize the informative positions relative to the hydropathy scale of amino acids, the hydropathy scale proposed by [57] was utilized and the average hydropathy of each column in the MSA was computed. Higher positive scores indicate that amino acids in that region have hydrophobic properties and are likely to be located in a transmembrane alpha-helix segment. The TCS score of each column in the alignment is noted on the hydropathy plot through color coding. Fig 4 shows diverse examples. The red shades correspond to the informative columns (TCS score ≥ 4), while the gray and white shades correspond to non-informative columns that are filtered out by TranCEP. In Fig 4(a) and 4(b), the regions with high positive average hydropathy values appear to be more informative compared to the ones with lower values. However, in Fig 4(c) and 4(d), the difference between the informative positions with high and low hydropathy values is not as clear.

To be able to measure the informative positions relative to different segments of the protein sequence, we divided the protein sequence positions into those in the TMS and those not in the TMS. Those in the TMS were divided into the interior one-third positions, and the remaining exterior positions in the TMS. The non-TMS positions were divided into those near a TMS, that is, within 10 positions, and the remaining positions far from a TMS. The location of the TMS was predicted using TOPCONS2 [58] to predict the *α*-helical TMS and PRED-TMBB2 [59] for *β*-barrel TMS. Table 7 shows a breakdown of where the informative positions, as determined by TCS, are located with respect TMS regions.

**Table 7:** Positional information.

Class	SeqLth	TMS	TMSLth	Positions		TMS			non-TMS		
				Num	%Seq	Num	Interior Num	Exterior Num	Num	Close Num	Far Num
Amino acid	535	10	214	340	60.91	173	59	114	153	85	68
Anion	632	10	199	480	68.95	180	61	119	278	98	180
Cation	644	7	148	443	61.29	131	44	87	287	72	215
Electron	352	3	57	281	41.59	51	17	34	124	28	96
Protein	592	2	38	350	32.04	26	9	17	136	20	116
Sugar	467	10	215	345	70.37	193	65	128	143	80	63
Other	580	7	155	419	60.23	129	43	86	249	69	180

The table presents information on the sites retained by the TCS filtering step. For each class of substrates in the dataset, the table presents the average sequence length (**SeqLth**), the average number of TMS regions (**TMS**), and the average total number of residues in the TMS regions (**TMSLth**). It also presents the average of the number of positions retained by the filtering (**Positions:Num**), and that number as a percentage of the total sequence length (**Positions:%Seq**). It notes the total number of sites that occur in the TMS regions (**TMS:Num**), and the non-TMS regions (**non-TMS:Num**). For the TMS regions, it presents the average number of sites that occur in the interior central one-third of the TMS regions (**TMS:Interior:Num**), and in the remaining exterior regions outside the central one-third of the TMS regions (**TMS:Exterior:Num**). For the non-TMS regions, it presents the average number of sites that occur close to the TMS regions (within 10 positions of the TMS) (**non-TMS:Close:Num**), and the remaining sites far from the TMS regions (**non-TMS:Far:Num**).

For instance, in Fig 4(b), 45.47% of the residues of the sequence with UniProt-ID P10970 are informative (i.e., correspond to informative columns in the alignment); thus 54.53% of this sequence is filtered out. In this case, the residues in the TMSs of this protein are indeed more informative, where 96.42% of them are informative. On the other hand, only 25% of the residues on non-TMSs are informative. However, this does not always hold true as in the sequence with UniProt-ID P0AAT4 in Fig 4(d), for example. Details of the sequences in the figure are presented in Table 8. Details of all the sequences in all of the examined segments are found in the open source repository.

**Table 8:** Examples of the informative residues distribution with respect to TMS and non-TMS.

UniProt-ID	SeqLth	TMS	TMSLth	Positions		TMS		non-TMS	
				Num	%Seq	Num	%Seq	Num	%Seq
P39515	158	4	80	113	71.51	73	91.25	40	51.28
P10870	884	12	252	402	45.47	243	96.42	159	25.15
P11551	438	12	252	252	57.53	148	58.73	94	55.91
P0AAT4	415	5	105	208	50.12	41	39.05	167	53.87

The table shows the details of Fig 4 individual sequences. The table presents the sequence length (**SeqLth**), the number of TMS regions (**TMS**), and the total number of residues in the TMS regions (**TMSLth**). It also presents the number of informative positions retained by the filtering (**Positions:Num**), and that number as a percentage of the total sequence length (**Positions:%Seq**). It notes the total number of informative sites that occur in the TMS regions (**TMS:Num**), and that number as a percentage of the total TMS length (**TMS:%Seq**). In addition, It notes the total number of informative sites that occur in the non-TMS regions (**non-TMS:Num**), and that number as a percentage of the total non-TMS length (**non-TMS:%Seq**).

Table 9 presents a pairwise comparison between informative positions in TMS and non-TMS regions. The sequences in *amino acid*, *anion*, *cation*, *sugar*, and *other*, substrate classes have significantly more informative positions in the TMS regions compared with non-TMS regions. However, the difference is not significant in the sequences that belong to *protein/mRNA* and *electron* substrates. This could be because these two classes have fewer TMSs per sequence, and TCS did not capture their conservation. The same difference in significance positions close to TMSs and positions far from TMSs is found, as shown in Table 10. In contrast, there is no difference between classes for interior and exterior segments of the TMSs, as presented in Table 11.

**Table 9:** Statistical analysis of informative position rates in TMS and non-TMS regions.

Class	TMS	non-TMS	P-value
Amino acid	74.61±27.28	50.06±21.61	<0.0001
Anion	82.16±30.35	60.94±26.93	<0.0001
Cation	76.51±34.41	55.71±28.63	<0.0001
Electron	37.62±44.12	41.44±43.56	0.050
Protein	32.66±39.83	30.92±35.30	0.414
Sugar	84.68±23.27	57.24±19.46	<0.0001
Other	67.53±38.21	54.52±31.17	<0.0001

All the data are reported as the sample mean ± the standard deviation (SD). The location of the TMS regions was predicted using TOPCONS2 [58] for the *α*-helical TMS and PRED-TMBB2 [59] for *β*-barrel TMS regions. There are statistically (P-value <0.0001) significant informative positions in TMS regions compared to non-TMS regions, in the sequences that belong to *amino acid*, *anion*, *cation*, *sugar*, and *other* classes.

**Table 10:** Statistical analysis of informative position rates close to TMS regions and far from TMS regions.

Class	Close	Far	P-value
Amino acid	62.59±24.73	39.08±23.08	<0.0001
Anion	72.53±28.28	53.86±28.19	<0.0001
Cation	66.20±32.40	51.35±28.91	<0.0001
Electron	36.67±44.05	41.67±43.76	0.035
Protein	32.38±39.47	29.96±34.54	0.313
Sugar	68.89±19.89	47.30±20.06	<0.0001
Other	62.84±35.31	48.91±30.90	<0.0001

All the data are reported as the sample mean ± the standard deviation (SD). For the non-TMS regions, there are statistically (P-value <0.0001) significant informative positions that occur close to the TMS regions (within 10 positions of the TMS) compared to other far regions, in the sequences that belong to *amino acid*, *anion*, *cation*, *sugar*, and *other* classes.

**Table 11:** Statistical analysis of informative position rates in the interior TMS regions and exterior TMS regions.

Class	Interior	Exterior	P-value
Amino acid	75.92±27.55	74.35±27.31	0.007
Anion	82.67±30.70	82.25±30.33	0.290
Cation	77.18±35.50	76.40±34.22	0.028
Electron	37.69±44.51	37.70±44.17	0.983
Protein	31.99±40.55	33.12±40.10	0.278
Sugar	86.16±24.51	84.37±22.96	0.011
Other	67.88±38.48	67.51±38.31	0.317

All the data are reported as the sample mean ± the standard deviation (SD). For the TMS regions, there is no difference between the informative positions in the interior central one-third of the TMS regions and the remaining exterior regions, in all sequences that belong to all substrate classes.

## Conclusion

We have developed a novel method TranCEP for *de novo* prediction of substrates for membrane transporter proteins that combines information based on amino acid composition, evolutionary information, and positional information. TranCEP incorporates first, the use of evolutionary information taking 120 similar sequences and constructing a multiple sequence alignment using TM-Coffee, second, the use of positional information by filtering to reliable positions as determined by TCS, and third, the use of pair amino acid composition. TranCEP achieved an overall MCC of 0.69, which is a 68% improvement over the state-of-the-art method TrSSP that uses the primary sequence alone. In addition, we evaluated the impact on performance of each factor: incorporating evolutionary information and filtering the unreliable positions. We observed that using amino acid composition alone does not obtain strong performance. The enhanced performance came mainly from incorporating evolutionary and positional information. We learned that certain positions in the alignment have greater significance, and identifying them helped to boost the performance.

## Supporting information

S1 File Five-fold cross-validation performance details. (PDF)

S2 File Leave-one-out cross-validation performance details. (PDF)

S3 File Independent testing performance details. (PDF)

S1 Table Manuscript tables (.xlsx). (XLSX)

S2 Table Training data (.xlsx). (XLSX)

S3 Table Testing data (.xlsx). (XLSX)
