# Supplementary material for: TranCEP: Predicting the substrate class of transmembrane transport proteins using compositional, evolutionary, and positional information
Source: PLoS One. 2020 Jan 14;15(1):e0227683. doi: 10.1371/journal.pone.0227683 (PMC6959595; doi:10.1371/journal.pone.0227683)
Supplement: S1 File — (PDF) [file pone.0227683.s001.pdf]

Five-Fold Cross-Validation Performance

1 Performance Plots

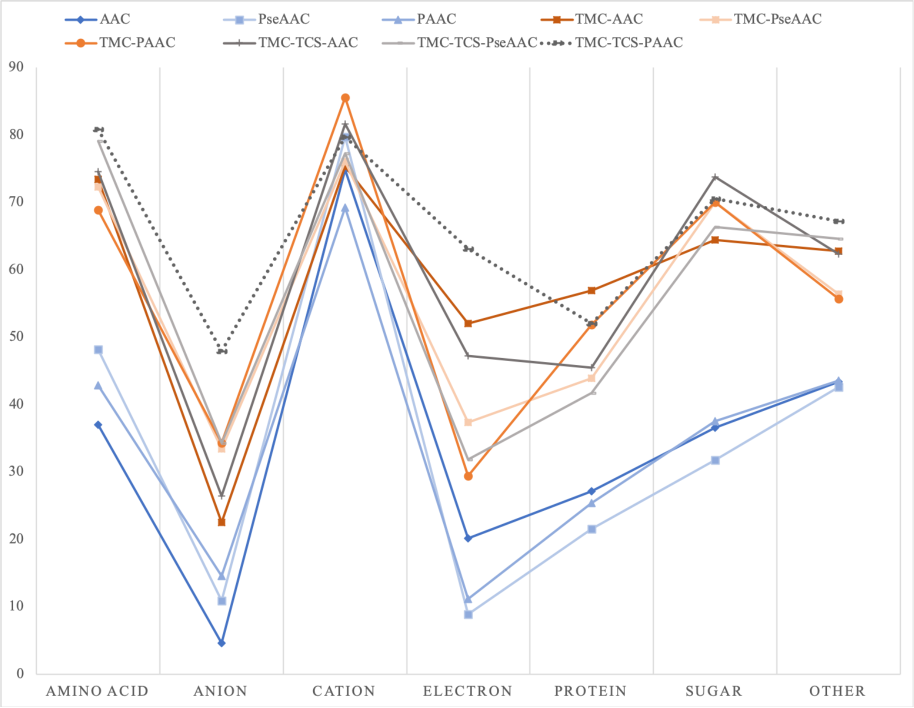

Figure 1: Sensitivity of different methods on different classes
The dotted line represents the performance of TranCEP, the TMC-TCS-PAAC method.

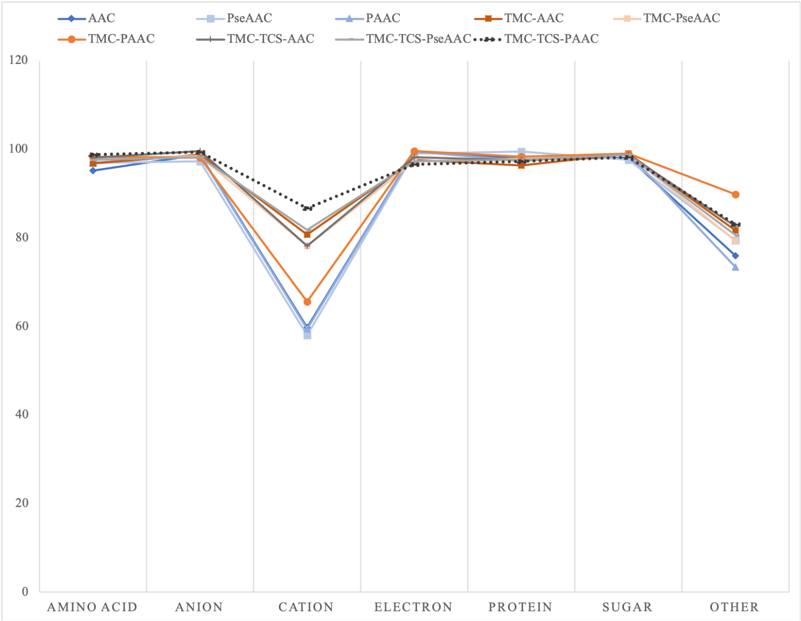

Figure 2: Specificity of different methods on different classes
The dotted line represents the performance of TranCEP, the TMC-TCS-PAAC method.

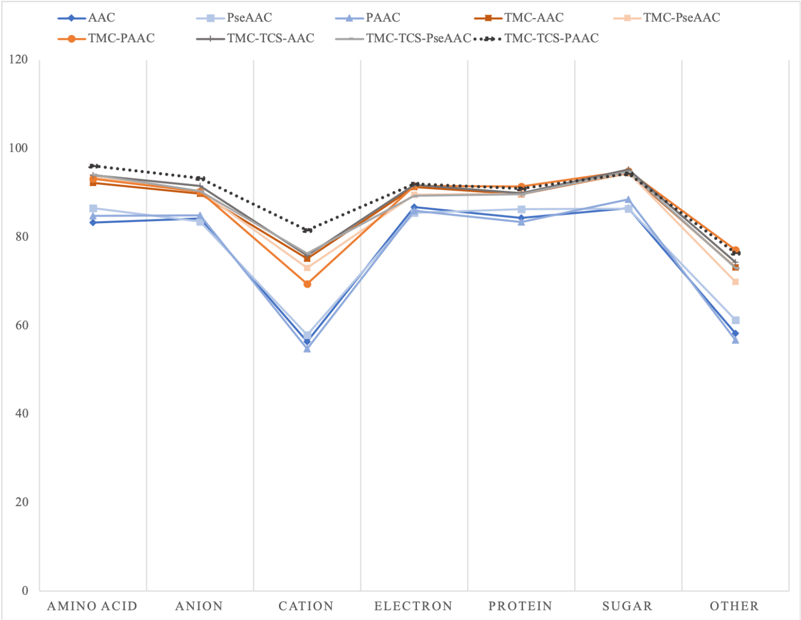

Figure 3: Accuracy of different methods on different classes
The dotted line represents the performance of TranCEP, the TMC-TCS-PAAC method.

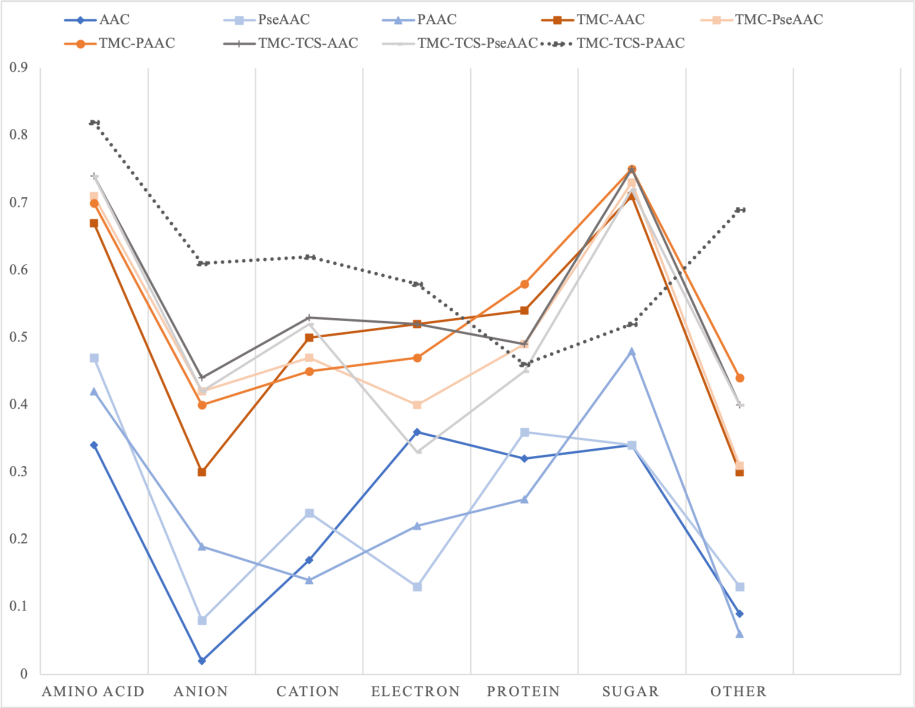

Figure 4: MCC of different methods on different classes
The dotted line represents the performance of TranCEP, the TMC-TCS-PAAC method.

2 Protein compositions

Class	Specificity	Sensitivity	Accuracy	MCC
**Amino acid**	95.22	37.00	83.28	0.34
**Anion**	98.76	4.61	84.20	0.02
**Cation**	59.79	74.83	56.35	0.17
**Electron**	99.45	20.16	86.81	0.36
**Protein**	97.90	27.13	84.32	0.32
**Sugar**	98.05	36.59	86.53	0.34
**Other**	75.90	43.37	58.30	0.09
**Overall**			45.38	0.22

Table 1: AAC cross-validation performance

Class	Specificity	Sensitivity	Accuracy	MCC
**Amino acid**	96.92	42.86	84.77	0.42
**Anion**	98.61	14.51	84.95	0.19
**Cation**	59.47	69.24	54.78	0.14
**Electron**	99.45	11.18	85.98	0.22
**Protein**	97.33	25.33	83.39	0.26
**Sugar**	99.03	37.55	88.58	0.48
**Other**	73.34	43.53	56.72	0.06
**Overall**			45.89	0.25

Table 2: PAAC cross-validation performance

Class	Specificity	Sensitivity	Accuracy	MCC
**Amino acid**	96.92	48.21	86.55	0.47
**Anion**	97.23	10.88	83.48	0.08
**Cation**	58.07	79.70	57.93	0.24
**Electron**	99.03	8.87	85.46	0.13
**Protein**	99.44	21.50	86.32	0.36
**Sugar**	97.65	31.74	86.47	0.34
**Other**	79.43	42.61	61.27	0.13
**Overall**			47.69	0.27

Table 3: PseAAC cross-validation performance

Class	Specificity	Sensitivity	Accuracy	MCC
**Amino acid**	96.76	73.43	92.23	0.67
**Anion**	98.75	22.46	89.84	0.30
**Cation**	80.70	75.35	75.10	0.50
**Electron**	97.66	51.98	91.30	0.52
**Protein**	96.35	56.93	89.72	0.54
**Sugar**	99.03	64.42	94.49	0.71
**Other**	81.68	62.75	73.21	0.3
**Overall**			63.08	0.50

Table 4: TMC-AAC cross-validation performance

Class	Specificity	Sensitivity	Accuracy	MCC
**Amino acid**	98.32	68.86	93.17	0.70
**Anion**	98.07	34.31	90.32	0.40
**Cation**	65.60	85.56	69.46	0.45
**Electron**	99.59	29.39	91.51	0.47
**Protein**	98.33	51.85	91.47	0.58
**Sugar**	99.03	69.98	94.97	0.75
**Other**	89.82	55.65	77.17	0.44
**Overall**			63.33	0.51

Table 5: TMC-PAAC cross-validation performance

Class	Specificity	Sensitivity	Accuracy	MCC
**Amino acid**	98.03	72.23	93.52	0.71
**Anion**	98.47	33.43	90.35	0.42
**Cation**	78.15	76.04	73.07	0.47
**Electron**	97.23	37.36	89.52	0.40
**Protein**	97.88	43.92	89.83	0.49
**Sugar**	98.75	70.00	94.64	0.73
**Other**	79.37	56.34	69.93	0.31
**Overall**			61.79	0.49

Table 6: TMC-PseAAC cross-validation performance

Class	Specificity	Sensitivity	Accuracy	MCC
**Amino acid**	98.05	74.52	94.05	0.74
**Anion**	99.58	26.44	91.50	0.44
**Cation**	78.15	81.66	75.80	0.53
**Electron**	98.19	47.19	91.81	0.52
**Protein**	97.48	45.47	89.88	0.49
**Sugar**	98.76	73.80	95.29	0.75
**Other**	82.60	62.30	74.27	0.40
**Overall**			65.13	0.58

Table 7: TCS-TMC-AAC cross-validation performance

Class	Specificity	Sensitivity	Accuracy	MCC
**Amino acid**	98.82	80.84	96.06	0.82
**Anion**	99.30	47.89	93.33	0.61
**Cation**	86.66	79.70	81.50	0.62
**Electron**	96.58	63.13	92.01	0.58
**Protein**	97.21	52.00	90.93	0.46
**Sugar**	98.19	70.51	94.39	0.52
**Other**	83.02	67.23	76.43	0.69
**Overall**			69.23	0.63

Table 8: TCS-TMC-PAAC cross-validation performance

Class	Specificity	Sensitivity	Accuracy	MCC
**Amino acid**	97.60	79.06	94.16	0.74
**Anion**	98.19	34.26	90.34	0.42
**Cation**	81.72	77.24	76.31	0.52
**Electron**	97.22	31.86	89.29	0.33
**Protein**	97.61	41.68	89.68	0.45
**Sugar**	98.72	66.35	94.82	0.72
**Other**	80.64	64.59	73.06	0.40
**Overall**			63.71	0.51

Table 9: TCS-TMC-PseAAC cross-validation performance
