# Supplementary material for: TranCEP: Predicting the substrate class of transmembrane transport proteins using compositional, evolutionary, and positional information
Source: PLoS One. 2020 Jan 14;15(1):e0227683. doi: 10.1371/journal.pone.0227683 (PMC6959595; doi:10.1371/journal.pone.0227683)
Supplement: S2 File — (PDF) [file pone.0227683.s002.pdf]

Leave-one-out Cross-Validation Performance

1 Performance Plots

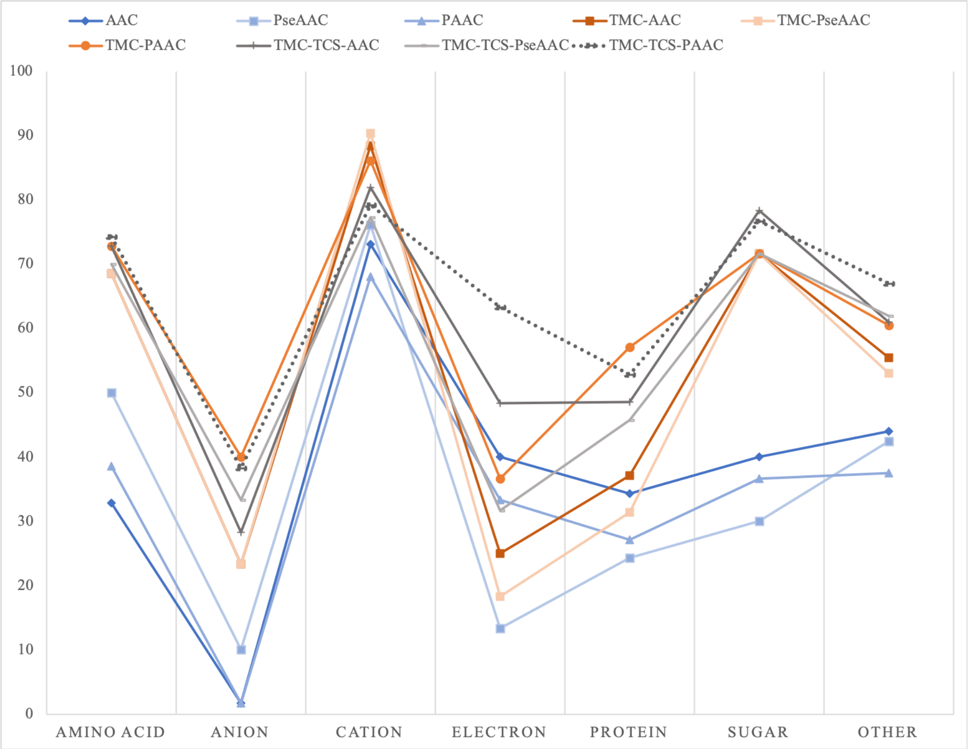

Figure 1: Sensitivity of different methods on different classes

The dotted line represents the performance of TranCEP, the TMC-TCS-PAAC method.

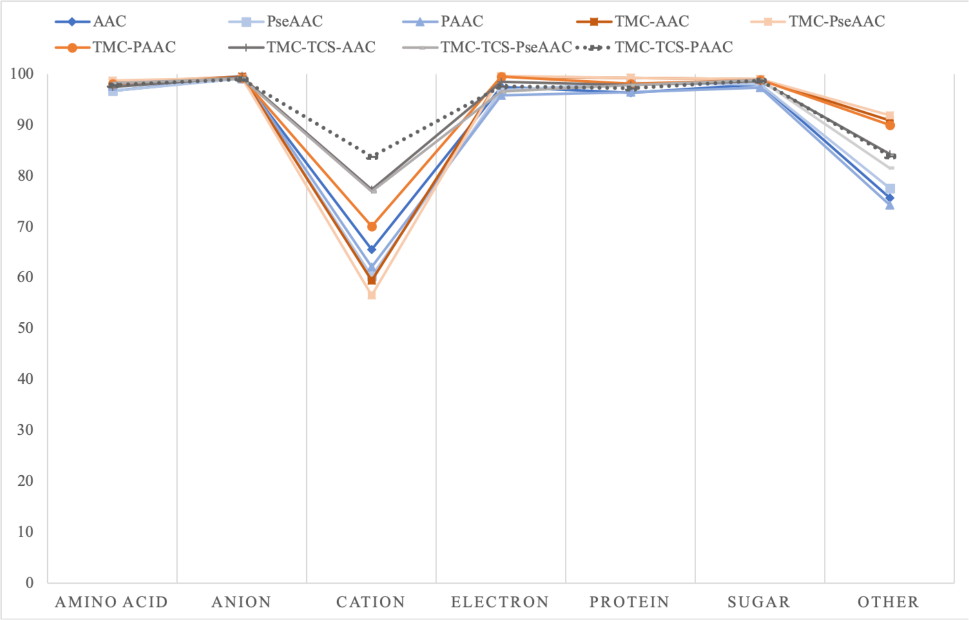

Figure 2: Specificity of different methods on different classes

The dotted line represents the performance of TranCEP, the TMC-TCS-PAAC method.

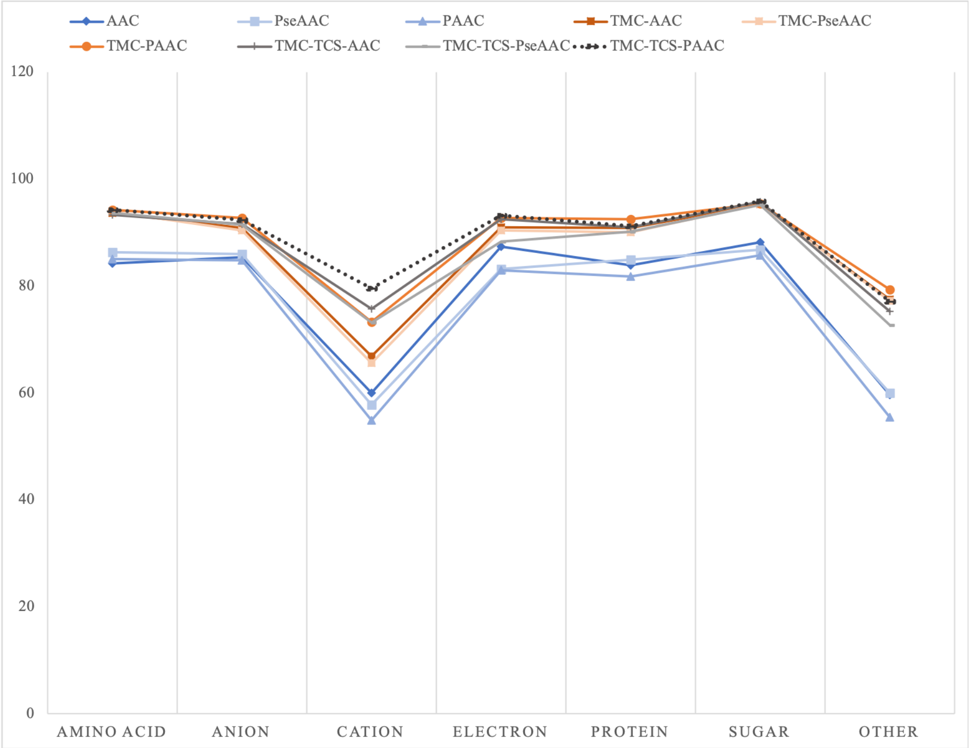

Figure 3: Accuracy of different methods on different classes

The dotted line represents the performance of TranCEP, the TMC-TCS-PAAC method.

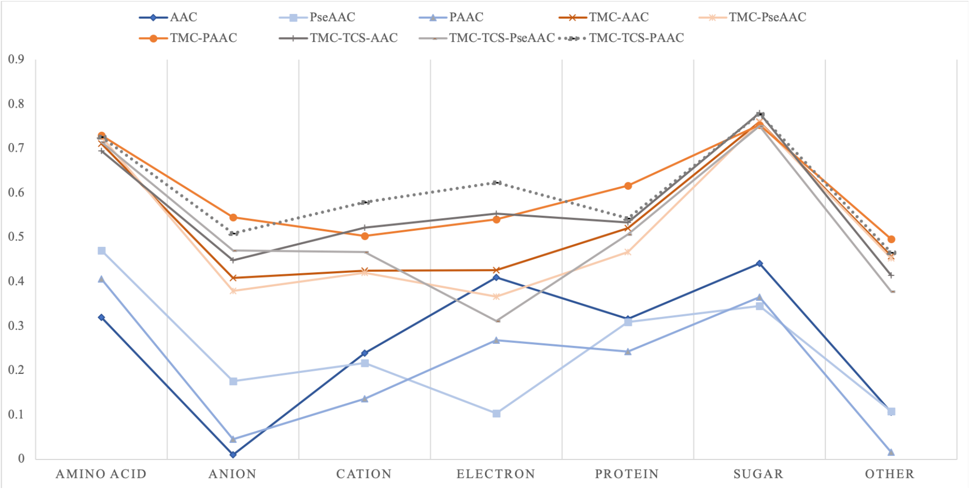

Figure 4: MCC of different methods on different classes

The dotted line represents the performance of TranCEP, the TMC-TCS-PAAC method.

2 Protein compositions

Class	Specificity	Sensitivity	Accuracy	MCC
**Amino acid**	96.76	32.86	84.23	0.32
**Anion**	99.31	1.67	85.39	0.01
**Cation**	65.58	73.08	60.03	0.24
**Electron**	97.50	40.00	87.38	0.41
**Protein**	96.34	34.29	83.86	0.32
**Sugar**	98.06	40.00	88.21	0.44
**Other**	75.69	44.00	59.65	0.11
**Overall**			47.95	0.26

Table 1: AAC LOOCV performance

Class	Specificity	Sensitivity	Accuracy	MCC
**Amino acid**	97.61	38.57	85.04	0.41
**Anion**	99.72	1.67	84.83	0.04
**Cation**	62.12	68.08	54.91	0.14
**Electron**	95.83	33.33	82.97	0.27
**Protein**	96.48	27.14	81.78	0.24
**Sugar**	97.36	36.67	85.68	0.37
**Other**	74.31	37.50	55.45	0.02
**Overall**			43.72	0.21

Table 2: PAAC LOOCV performance

Class	Specificity	Sensitivity	Accuracy	MCC
**Amino acid**	96.76	50.00	86.35	0.47
**Anion**	99.17	10.00	85.95	0.18
**Cation**	60.38	76.15	57.80	0.22
**Electron**	96.94	13.33	83.22	0.10
**Protein**	98.31	24.29	84.95	0.31
**Sugar**	98.06	30.00	86.76	0.34
**Other**	77.59	42.50	59.97	0.11
**Overall**			47.05	0.25

Table 3: PseAAC LOOCV performance

Class	Specificity	Sensitivity	Accuracy	MCC
**Amino acid**	98.45	68.57	93.65	0.71
**Anion**	99.58	23.33	90.86	0.41
**Cation**	59.42	88.46	66.90	0.43
**Electron**	99.58	25.00	91.03	0.43
**Protein**	99.30	37.14	90.86	0.52
**Sugar**	99.03	71.67	95.30	0.76
**Other**	90.86	55.50	77.42	0.46
**Overall**			62.44	0.53

Table 4: TMC-AAC LOOCV performance

Class	Specificity	Sensitivity	Accuracy	MCC
**Amino acid**	98.17	72.86	94.25	0.73
**Anion**	99.31	40.00	92.76	0.54
**Cation**	70.19	86.15	73.32	0.50
**Electron**	99.58	36.67	92.76	0.54
**Protein**	98.17	57.14	92.43	0.62
**Sugar**	98.89	71.67	95.45	0.75
**Other**	90.00	60.50	79.31	0.50
**Overall**			67.31	0.60

Table 5: TMC-PAAC LOOCV performance

Class	Specificity	Sensitivity	Accuracy	MCC
**Amino acid**	98.73	68.57	93.92	0.73
**Anion**	99.31	23.33	90.38	0.38
**Cation**	56.54	90.39	65.62	0.42
**Electron**	99.72	18.33	90.38	0.37
**Protein**	99.30	31.43	90.04	0.47
**Sugar**	99.03	71.67	95.23	0.76
**Other**	91.90	53.00	77.26	0.45
**Overall**			61.41	0.51

Table 6: TMC-PseAAC LOOCV performance

Class	Specificity	Sensitivity	Accuracy	MCC
**Amino acid**	97.47	72.86	93.27	0.70
**Anion**	99.44	28.33	91.61	0.45
**Cation**	77.31	81.92	75.66	0.52
**Electron**	98.47	48.33	92.43	0.55
**Protein**	97.89	48.57	90.96	0.53
**Sugar**	98.61	78.33	95.71	0.78
**Other**	84.31	61.00	75.22	0.41
**Overall**			65.77	0.56

Table 7: TMC-TCS-AAC LOOCV performance

Class	Specificity	Sensitivity	Accuracy	MCC
**Amino acid**	97.89	74.29	94.20	0.73
**Anion**	99.03	38.33	92.41	0.51
**Cation**	83.85	79.23	79.53	0.58
**Electron**	97.64	63.33	93.22	0.62
**Protein**	97.32	52.86	91.16	0.54
**Sugar**	98.75	76.67	95.89	0.78
**Other**	83.97	67.00	77.12	0.47
**Overall**			68.72	0.60

Table 8: TMC-TCS-PAAC LOOCV performance

Class	Specificity	Sensitivity	Accuracy	MCC
**Amino acid**	98.31	70.00	93.67	0.71
**Anion**	99.17	33.33	91.39	0.47
**Cation**	76.92	77.31	73.16	0.47
**Electron**	96.67	31.67	88.25	0.31
**Protein**	97.89	45.71	90.20	0.51
**Sugar**	98.89	71.67	95.13	0.75
**Other**	81.55	62.00	72.73	0.38
**Overall**			62.56	0.51

Table 9: TMC-TCS-PseAAC LOOCV performance
