# Supplementary material for: TranCEP: Predicting the substrate class of transmembrane transport proteins using compositional, evolutionary, and positional information
Source: PLoS One. 2020 Jan 14;15(1):e0227683. doi: 10.1371/journal.pone.0227683 (PMC6959595; doi:10.1371/journal.pone.0227683)
Supplement: S3 File — (PDF) [file pone.0227683.s003.pdf]

Independent Testing Performance

1 Performance Plots

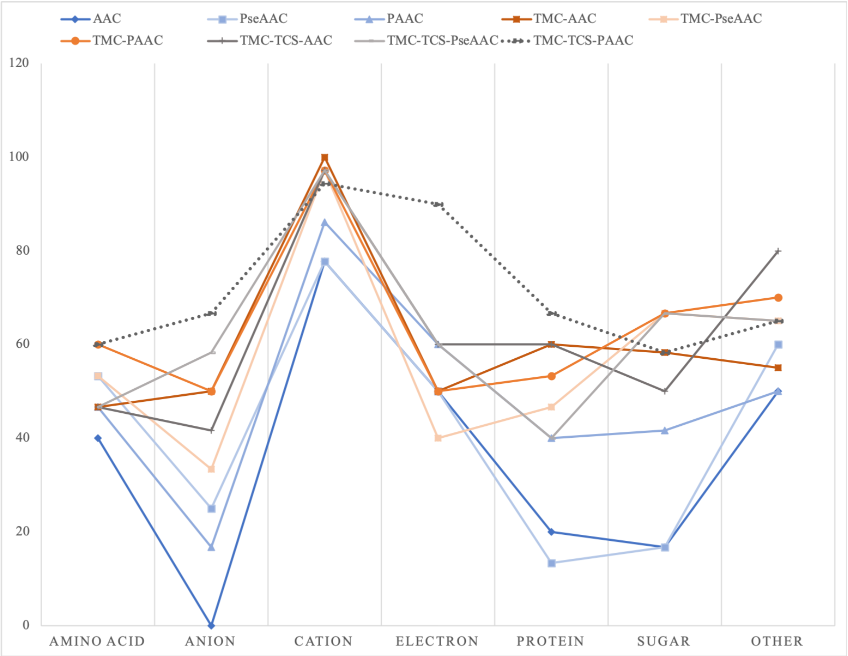

Figure 1: Sensitivity of different methods on different classes

The dotted line represents the performance of TranCEP, the TMC-TCS-PAAC method.

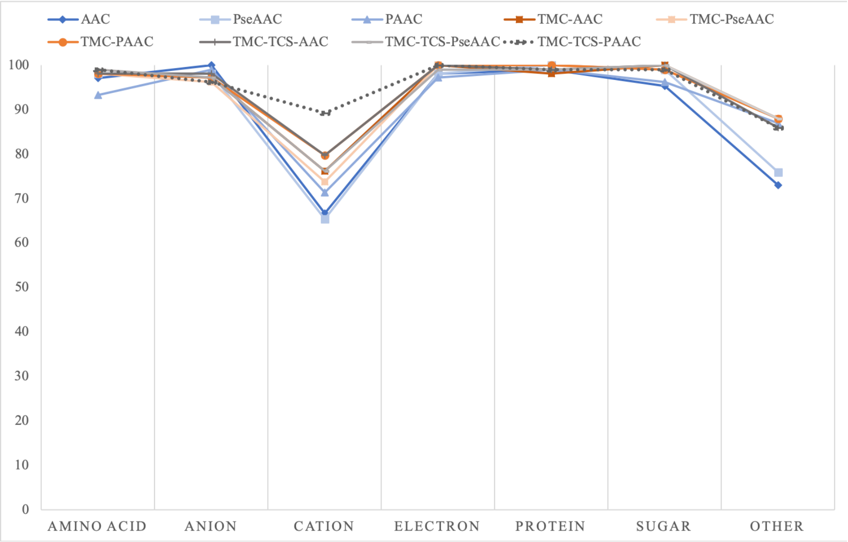

Figure 2: Specificity of different methods on different classes

The dotted line represents the performance of TranCEP, the TMC-TCS-PAAC method.

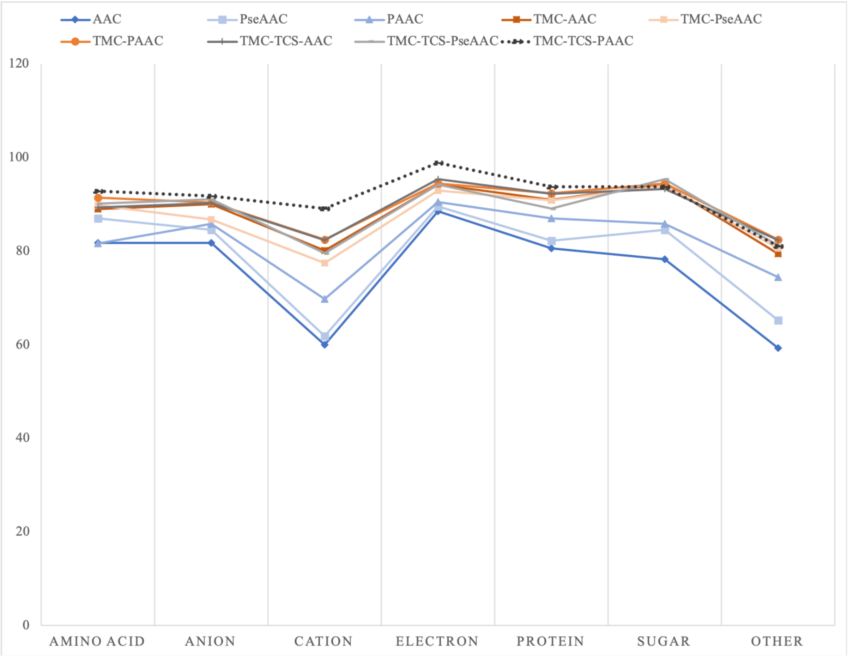

Figure 3: Accuracy of different methods on different classes

The dotted line represents the performance of TranCEP, the TMC-TCS-PAAC method.

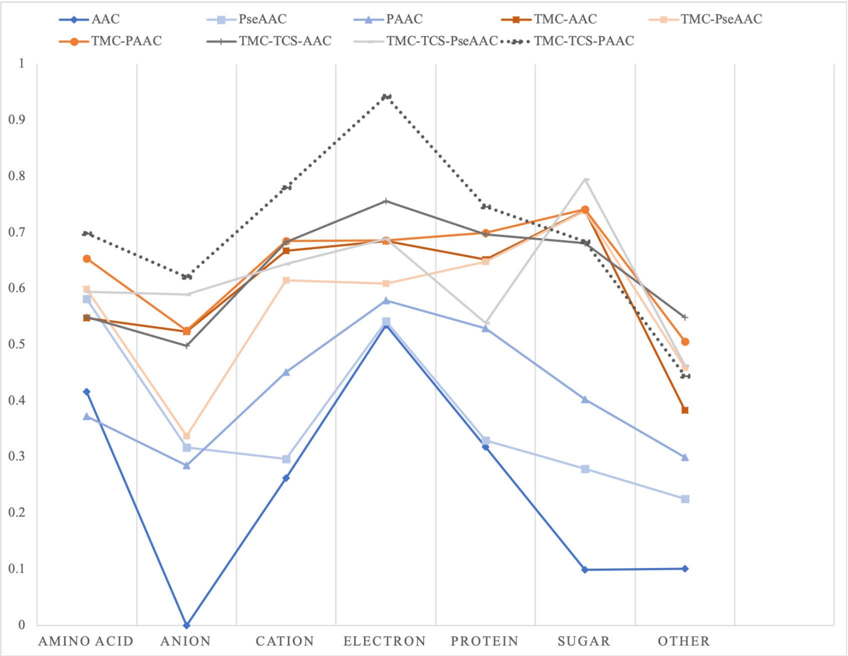

Figure 4: MCC of different methods on different classes

The dotted line represents the performance of TranCEP, the TMC-TCS-PAAC method.

2 Confusion matrix

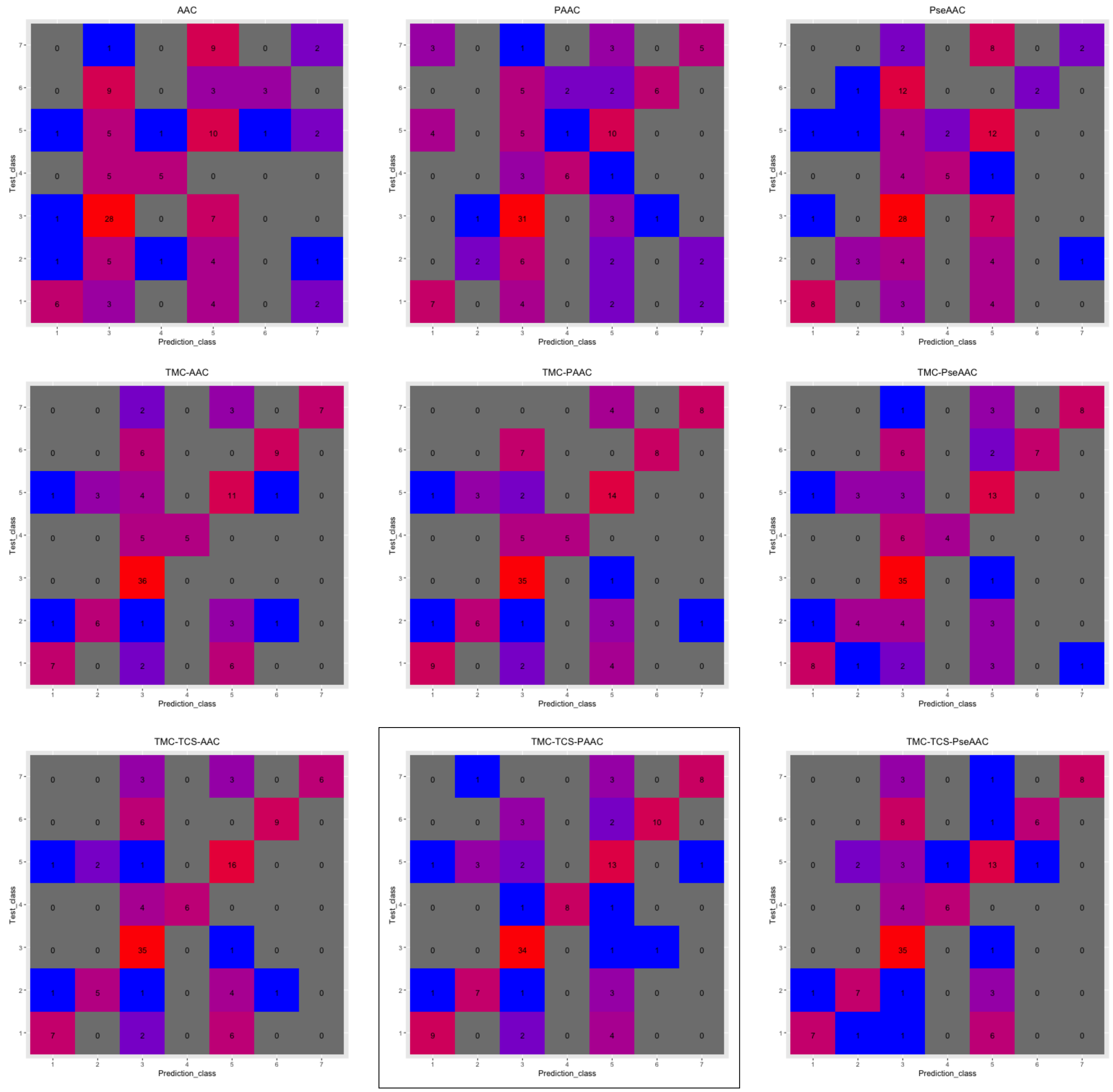

Figure 5: Confusion matrices

This figure presents the number of proteins in an actual substrate class that are predicted by different SVM models with different methods. The bordered matrix indicates the proposed tool prediction. The class can belong to one of the following seven categories (ordered alphabetically): [1] amino [2] anion [3] cation [4] electron [5] other [6] protein [7] sugar.

3 Protein compositions

Class	Specificity	Sensitivity	Accuracy	MCC
**Amino acid**	97.14	40.00	81.82	0.42
**Anion**	100.00	0.00	81.82	
**Cation**	66.67	77.78	60.00	0.26
**Electron**	98.18	50.00	88.53	0.54
**Protein**	99.05	20.00	80.60	0.32
**Sugar**	95.37	16.67	78.26	0.10
**Other**	73.00	50.00	59.34	0.10
**Overall**			45.00	

Table 1: AAC independant testing performance

Class	Specificity	Sensitivity	Accuracy	MCC
**Amino acid**	93.33	46.67	81.71	0.37
**Anion**	99.07	16.67	85.90	0.28
**Cation**	71.43	86.11	69.79	0.45
**Electron**	97.27	60.00	90.54	0.58
**Protein**	99.05	40.00	87.01	0.53
**Sugar**	96.30	41.67	85.90	0.40
**Other**	87.00	50.00	74.44	0.30
**Overall**			55.83	0.42

Table 2: PAAC independant testing performance

Class	Specificity	Sensitivity	Accuracy	MCC
**Amino acid**	98.09	53.33	86.96	0.58
**Anion**	98.15	25.00	84.51	0.32
**Cation**	65.48	77.78	61.86	0.30
**Electron**	98.18	50.00	89.55	0.54
**Protein**	100.00	13.33	82.19	0.33
**Sugar**	99.07	16.67	84.51	0.28
**Other**	76.00	60.00	65.22	0.23
**Overall**			50.00	0.37

Table 3: PseAAC independant testing performance

Class	Specificity	Sensitivity	Accuracy	MCC
**Amino acid**	98.09	46.67	89.01	0.55
**Anion**	97.22	50.00	90.00	0.52
**Cation**	76.19	100.00	80.20	0.67
**Electron**	100.00	50.00	94.19	0.68
**Protein**	98.09	60.00	91.01	0.65
**Sugar**	100.00	58.33	94.19	0.74
**Other**	88.00	55.00	79.41	0.38
**Overall**			67.50	0.60

Table 4: TMC-AAC independant testing performance

Class	Specificity	Sensitivity	Accuracy	MCC
**Amino acid**	98.09	60.00	91.40	0.65
**Anion**	97.22	50.00	90.43	0.53
**Cation**	79.76	97.22	82.52	0.69
**Electron**	100.00	50.00	94.44	0.69
**Protein**	100.00	53.33	92.39	0.70
**Sugar**	99.07	66.67	94.44	0.74
**Other**	88.00	70.00	82.52	0.51
**Overall**			70.83	0.64

Table 5: TMC-PAAC independant testing performance

Class	Specificity	Sensitivity	Accuracy	MCC
**Amino acid**	98.09	53.33	89.77	0.60
**Anion**	96.30	33.33	86.81	0.34
**Cation**	73.81	97.22	77.45	0.61
**Electron**	100.00	40.00	92.94	0.61
**Protein**	100.00	46.67	90.81	0.65
**Sugar**	99.07	66.67	94.05	0.74
**Other**	88.00	65.00	80.61	0.46
**Overall**			65.83	0.57

Table 6: TMC-PseAAC independant testing performance

Class	Specificity	Sensitivity	Accuracy	MCC
**Amino acid**	98.09	46.67	89.36	0.55
**Anion**	98.15	41.67	90.32	0.50
**Cation**	79.76	97.22	82.35	0.68
**Electron**	100.00	60.00	95.45	0.76
**Protein**	99.05	60.00	92.31	0.70
**Sugar**	100.00	50.00	93.33	0.68
**Other**	86.00	80.00	82.35	0.55
**Overall**			70.00	0.63

Table 7: TMC-TCS-AAC independant testing performance

Class	Specificity	Sensitivity	Accuracy	MCC
**Amino acid**	98.10	60.00	91.75	0.66
**Anion**	96.30	58.33	90.82	0.56
**Cation**	89.29	94.44	89.00	0.78
**Electron**	100.00	80.00	97.80	0.88
**Protein**	99.07	66.67	93.68	0.75
**Sugar**	99.07	66.67	94.68	0.74
**Other**	86.00	65.00	80.91	0.44
**Overall**			74.17	0.69

Table 8: TMC-TCS-PAAC independant testing performance

Class	Specificity	Sensitivity	Accuracy	MCC
**Amino acid**	99.05	46.67	90.11	0.59
**Anion**	97.22	58.33	91.11	0.59
**Cation**	76.19	97.22	79.61	0.64
**Electron**	99.09	60.00	94.25	0.69
**Protein**	99.05	40.00	89.13	0.54
**Sugar**	100.00	66.67	95.35	0.80
**Other**	88.00	65.00	81.19	0.46
**Overall**			68.33	0.62

Table 9: TMC-TCS-PseAAC independant testing performance
